# Feature Ranking by Variational Dropout for Classification Using Thermograms from Diabetic Foot Ulcers

**DOI:** 10.3390/s23020757

**Published:** 2023-01-09

**Authors:** Abian Hernandez-Guedes, Natalia Arteaga-Marrero, Enrique Villa, Gustavo M. Callico, Juan Ruiz-Alzola

**Affiliations:** 1 Instituto Universitario de Investigaciones Biomédicas y Sanitarias (IUIBS), Universidad de Las Palmas de Gran Canaria, 35016 Las Palmas de Gran Canaria, Spain; 2 Instituto Universitario de Microelectrónica Aplicada (IUMA), Universidad de Las Palmas de Gran Canaria, 35017 Las Palmas de Gran Canaria, Spain; 3 Grupo Tecnología Médica IACTEC, Instituto de Astrofísica de Canarias (IAC), 38205 San Cristóbal de La Laguna, Spain; 4 Departamento de Señales y Comunicaciones, Universidad de Las Palmas de Gran Canaria, 35016 Las Palmas de Gran Canaria, Spain

**Keywords:** thermography, infrared, deep learning, feature extraction, diabetic foot

## Abstract

Diabetes mellitus presents a high prevalence around the world. A common and long-term derived complication is diabetic foot ulcers (DFUs), which have a global prevalence of roughly 6.3%, and a lifetime incidence of up to 34%. Infrared thermograms, covering the entire plantar aspect of both feet, can be employed to monitor the risk of developing a foot ulcer, because diabetic patients exhibit an abnormal pattern that may indicate a foot disorder. In this study, the publicly available INAOE dataset composed of thermogram images of healthy and diabetic subjects was employed to extract relevant features aiming to establish a set of state-of-the-art features that efficiently classify DFU. This database was extended and balanced by fusing it with private local thermograms from healthy volunteers and generating synthetic data via synthetic minority oversampling technique (SMOTE). State-of-the-art features were extracted using two classical approaches, LASSO and random forest, as well as two variational deep learning (DL)-based ones: concrete and variational dropout. Then, the most relevant features were detected and ranked. Subsequently, the extracted features were employed to classify subjects at risk of developing an ulcer using as reference a support vector machine (SVM) classifier with a fixed hyperparameter configuration to evaluate the robustness of the selected features. The new set of features extracted considerably differed from those currently considered state-of-the-art but provided a fair performance. Among the implemented extraction approaches, the variational DL ones, particularly the concrete dropout, performed the best, reporting an F1 score of 90% using the aforementioned SVM classifier. In comparison with features previously considered as the state-of-the-art, approximately 15% better performance was achieved for classification.

## 1. Introduction

Diabetes mellitus is a chronic disease whose global prevalence was estimated to be 10.5% (536.6 million people) in 2021, which is expected to rise to 12.2% (783.2 million) in 2045 according to the International Diabetes Federation [1]. Diabetic foot ulcers (DFUs) constitute a long-term and common complication derived from diabetes [2,3] with an estimated global prevalence of roughly 6.3% [4] and a lifetime incidence of between 19% and 34% for the diabetic population [5]. Ulcers represent the most frequently recognized and highest risk factor, because a possible infection of the wound often results in the amputation of the foot or lower limb. Worldwide, it is estimated that a limb is amputated every 20 s due to diabetes [6]. Furthermore, the recurrence rate of DFU is high and varies widely among different regions. The recurrence rates remain about 60% after three years [5], although these figures have been updated and, as of 2019, the recurrence rate estimation was 22.1% per person-year (py) [7]. The lowest recurrence rate was roughly 16.9% per py in Africa, while the highest was 24.9% per py in Europe [7].

These complications can be avoided, reduced, or substantially delayed by early detection, assessment, diagnosis, and tailored treatment [2,8]. DFU detection by machine learning (ML) or deep learning (DL) approaches is mainly focused on the already formed ulcer [9,10]. A large public dataset, composed of 4000 images with ground truth labeling, was released for the Diabetic Foot Ulcers Grand Challenge (DFUC 2020) aiming to improve the detection accuracy in a real-world scenario and to accelerate the development of innovative approaches [6]. In addition, extensive literature can be found for DFU localization and detection [11], as well as wound classification [12,13,14]. Furthermore, remote, noncontact, and automated DFU detection may be plausible using mobile and cloud technologies [6].

Alternatively, identifying the underlying conditions that sustain skin and tissue damage at an early stage, previous to the onset of superficial wounds, is an emerging area of research [15,16,17,18]. Early diagnosis is extremely valuable for any pathology, particularly one that can prevent a fatal outcome, as in the case of the present application. Infrared thermography has demonstrably established itself as a complementary tool for the early identification of superficial tissue damage. Real-time visualization of plantar temperature distribution is provided while the surface to be measured remains intact [3]. Thus, the entire plantar aspect of both feet can be conveniently analyzed in a very short time with great sensitivity and specificity, putting forward thermography as a suitable technique for diabetic neuropathy screening programs [19]. Nevertheless, the heat pattern of the plantar aspect of the feet and its association with diabetic foot pathologies are subtle and often nonlinear [20]. Thus, the interpretation of plantar thermograms requires the development of computer-aided eiagnosis (CAD) systems that do not rely on subjective interpretations or inherent limitations of human visual perception. Consequently, interobserver variability and workload may be decreased, whereas CAD systems may outperform clinicians regarding cost, accuracy, and speed, thus leading to an enhanced level of medical care [3].

Ideally, these CAD systems should classify subjects at risk of developing an ulcer from a single thermogram containing the plantar aspect of both feet and, if possible, quantify the severity of the lesion. Previous attempts proposed quantitative parameters for detecting thermal changes based on the varying temperature distribution exhibited by diabetic subjects in comparison with healthy ones [3]. Recently, the importance of early detection and gaps regarding performance accuracy were brought into focus, resulting in the development of an unsupervised approach for severity stratification [18]. Several features based on infrared thermography are proposed in the state-of-the-art methods for identifying foot disorders. Additionally, there is an interest in detecting features that are relevant for the detection of DFU [18]; different methods for feature selection are being explored.

Feature selection is a field of statistical multivariate and ML methods that reduces the number of input variables. The main objective is to find an optimal subset from the input variables set, *S*, that causes an improvement, for instance, in the classifiers by reducing the amount of redundant input data. This provides classifiers with a better cost–performance ratio. At the same time, it improves the interpretability of data, which are commonly high-dimensional [21].

Feature selection methods can be traditionally categorized into the following classes: filter, wrapper, and embedded methods. Filter methods consist of a preprocessing step that removes irrelevant features based on a per-feature relevance score [21,22,23,24]. The wrapper methods are those in which, after defining the searching subspace (all possible variable subsets) and applying a model as a black box, a search and evaluation strategy is carried out to obtain the optimal selection of variables or features [21,25]. These methods are computationally expensive and especially demanding in DL models [26]. Finally, embedded methods incorporate variable selection during the training process, employing a regularization for reducing the number of variables used during classification [21]. The least absolute shrinkage and selection operator (LASSO) regularization technique [27] is the most popular embedded method, whose objective function is constrained by an $L_{1}$ normalization. LASSO is widely used [28,29] but its main limitation is the restriction to linear functions.

In this study, following previous approaches to determine relevant features, variational dropout [30,31] was used as a feature selection embedded method for reducing the state-of-the-art variables used for DFU detection based on infrared thermograms. In addition, a new approach based on selecting the features in coincidence among the different feature selection methods was designed. The new set of features extracted was employed as input for a support vector machine (SVM) [32] classifier. The SVM classifier was used as a reference, with the aim of assessing the performance of these features. Finally, for comparison purposes, features previously reported as state-of-the-art were also fed to the classifier.

## 2. Material and Methods

### 2.1. Variational Dropout

Dropout [33], or binary dropout, is a regularization technique commonly used in DL models to reduce overfitting. This mechanism consists of applying multiplicative Bernoulli noise for each hidden unit in the neural network, i.e., it is a feed-forward operation described as:

(1) $$\begin{array}{cc} \; & z∼Bern(\rho ),\\ \; & \hat{\theta }=\theta ⊙z,\\ \; & y=\sigma (\hat{\theta }x+b), \end{array}$$

where *x* denotes the vector of inputs into the layer; θ and *b* are the weights and biases in the layer, respectively; σ is a nonlinear activation function, such as the sigmoid function; and ⊙ is a Hadamard dot product. The ρ value from the Bernoulli distribution, Bern(.), also known as the dropout rate value, is a hyperparameter and represents the probability of an element being zeroed.

The Gaussian dropout is an alternative to the aforementioned binary dropout. In this case, the Gaussian dropout defines the multiplicative noise, such as $r∼N(1,\frac{\rho }{1-\rho })$. Srivastava et al. reported that it has similar performance to the binary dropout with a dropout rate value ρ [33].

Variational dropout is a reinterpretation of the dropout with continuous noise, r∼N(1,α) as a variational method proposed by Kingma et al. [30]. In this case, α is a variational parameter of the model instead of a hyperparameter as in the Gaussian dropout. Using multiplicative noise is equivalent to putting noise on the weights so that the posterior distribution of the weights is given by $q_{\phi }\left(w_{i,j}\right)=N\left(\theta_{i,j},\alpha \theta_{i,j}^{2}\right)$. However, the application of noise in weight $w_{i,j}$ si gnificantly increases the variance of the gradients. For this reason, authors proposed the reparameterization trick [30,34] so the weights are sampled as:

(2) $$\begin{array}{cc} \; & w_{i,j}=f\left(\phi_{i,j},\epsilon_{i,j}\right)=\theta_{i,j}\left(1+\sqrt{\alpha }\epsilon_{i,j}\right)\\ \; & \epsilon_{i,j}∼N\left(0,1\right) \end{array}$$

where ϕ=(θ,α) are the variational parameters, and ϵ is the noise applied on the weights. The reparameterization trick leads to an estimator that has lower variance [30].

An alternative to variational dropout proposed by Kingma et al. is the concrete dropout [31], which is a variational approach to the original binary dropout. The Bernoulli distribution is problematic due to its discrete nature and does not allow efficient optimization based on gradients. For this reason, Gal et al. proposed replacing this discrete distribution with a continuous relaxation of the same, a concrete distribution relaxation [35]:

(3) $$\begin{array}{cc} \; & z_{i,j}=f\left(\phi ,\epsilon_{i,j}\right)=\sigma \left(\frac{1}{t}\left(\log\rho -\log\left(1-\rho \right)+\log\epsilon_{i,j}-\log\left(1-\epsilon_{i,j}\right)\right)\right)\\ \; & \epsilon_{i,j}∼U\left(0,1\right) \end{array}$$

where σ is a sigmoid function, t∈[0,1] is known as temperature, and ϕ=(ρ) is the variational parameter.

#### 2.1.1. Stochastic Variational Inference

In variational inference, given two random variables *X* and *Z*, p(Z|X) is approximated by a parametric distribution $q_{\phi }\left(Z\right)$, and the quality of this approximation is measured by the Kullback–Leibler divergence, $D_{KL}(q_{\phi }\left(Z\right)||p\left(Z|X\right))$. In this way, the optimal values for variational parameters ϕ are defined by the evidence lower bound [36]:

(4) $$L\left(\phi \right)=L\left(\phi \right)-D_{KL}(q_{\phi }\left(z\right)||p\left(z\right))$$

where L(ϕ) is the expected log-likelihood, which, in practice, can be interpreted as the cross-entropy loss function, and $D_{KL}(q_{\phi }\left(z\right)||p\left(z\right))$ works as a regularization term.

However, using complex models such as deep neural networks, Equation (4) is intractable; thus, the evidence lower bound and its gradient cannot be exactly computed. By using the reparameterization trick, where z=f(ϕ,ϵ), Kingma et al. [34] proposed an estimator of the marginal log-likelihood of the full dataset, based on mini-batches, named the stochastic gradient variational Bayes (SGVB) estimator:

(5) $$L^{SGVB}\left(\theta ,\phi ;X^{\left(m\right)}\right)=\frac{N}{M}\sum_{i=1}^{M}\log p\left({\tilde{y}}_{i}|x_{i},f\left(\phi ,\epsilon_{i}\right)\right)-D_{KL}(q_{\phi }\left(z\right)||p\left(z\right))$$

where $X^{m}\in \left\{x_{1},x_{2},\ldots x_{M}\right\}$ is a mini-batch of *M* random samples, from the full dataset *X*, composed of *N* samples.

#### 2.1.2. Feature Selection by Variational Dropout

**Figure 1:**
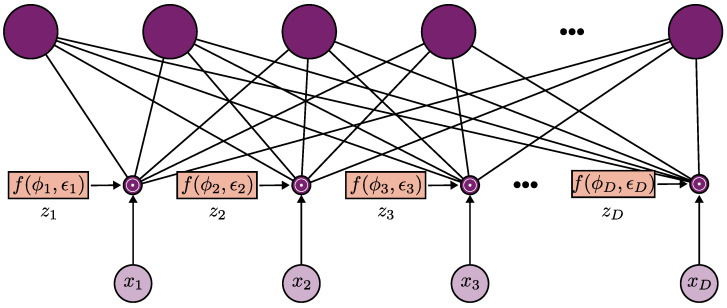
Dropout as a feature selection method. The variational parameter $\phi_{d}$, where d∈[1..D], controls the probability of the input feature $x_{d}$ to be dropped out, that is, an estimate of the noise level of $x_{d}$. The variational parameters are used for feature ranking.

The variational approach of the dropout can be interpreted as an embedded method for feature selection. Let $X\in R^{D}$ be the input domain of a target domain Y; the embedded method aims to obtain a subset $X_{S}\subset X$, i.e., a sparse representation where S<D. Taking this into account, the training objective is defined as follows:

(6) $$\underset{\theta ,s}{\mathrm{argmin}}L\left(f\left(X⊙s;\theta \right),Y\right)$$

where $s={\left\{0,1\right\}}^{D}$ is the vector indicating the *S* variables selected. Note that in the relationship with binary dropout, Equation (1), is appreciable. In this case, variational dropout consists of applying a dropout layer in the input layer as a feature selector. Figure 1 illustrates the proposed method employing variational dropout as a feature selector embedded method.

However, it is possible to use dropout with continuous noise for feature selection. Molchanov et al. [37] exhibited that variational dropout (from this point onwards, variational dropout will be used to refer to the dropout proposed by Kingma [30]) proposed by Kingma et al. [34] led to extremely sparse solutions. In Gaussian dropout, the case of α→∞ corresponds to a binary dropout where ρ=1 (recall $\alpha =\frac{\rho }{1-\rho }$). As the authors pointed out, an infinitely large α in the variational dropout corresponds to applying an infinitely large multiplicative noise in θ, concluding that this weight is absolutely random, as seen in Equation (2). In this case, the α variational parameter might be considered a feature ranking. The idea to force the sparse representation is to use a threshold τ for deactivating the features that are highly noisy.

Regarding concrete dropout, there is a limitation that prevents its use as a feature selection method: the regularization $D_{KL}$ in Equation (5). Thus, Gal et al. defined $D_{KL}$ as follows [31]:

(7) $$D_{KL}(q_{\phi }\left(w\right)||p\left(w\right))=\kappa_{\theta }{\left||\theta |\right|}^{2}-\kappa_{\rho }H\left(\rho \right)$$

where $\kappa_{\theta }$ and $\kappa_{\rho }$ are regularization factors for θ and ρ, respectively; and H(ρ) defines Shannon’s entropy of a Bernoulli random variable with probability ρ. The hyperparameters $\kappa_{\theta }$ and $\kappa_{\rho }$ have a specific relation [31], which is not specified here, because it is out of the scope of the present study. As can be observed in Equation (7), $\kappa_{\theta }$ forces θ to stay close to 0, and $\kappa_{\rho }$ pushes the dropout probability towards ρ=0.5, which is the point of maximum entropy of the distribution. Taking this into consideration, the variational parameter ρ would tend to be in ρ∈[0,0.5]. Nevertheless, it is recommended to force unnecessary features to values close to one.

Concrete dropout is a practical method for applying $l_{0}$ regularization that solves the aforementioned problem. Chang et al. [38] proposed penalizing the number of features not dropped-out, but its performance was highly dependent on the regularization factor. Louizos et al. [39] used a hard-concrete distribution that forces values between 0 and 1. Yamada et al. [40] proposed a Gaussian-based continuous relaxation of the Bernoulli distribution. All these approaches have the same purpose, which is to penalize the number of features used. For this reason, in order to use concrete dropout as a feature selector, Equation (5) is replaced with the following loss function:

(8) $$L\left(\theta ,\phi ;X^{\left(m\right)}\right)=\frac{1}{M}\sum_{i=1}^{M}\log p\left({\tilde{y}}_{i}|x_{i}⊙z_{i}\right)+\frac{1}{D}\sum_{j=1}^{D}\left(\Phi \left(\rho \right)\right)$$

where Φ(.) is the cumulative distribution function (CDF), and $z_{i}=f\left(\phi_{i},\epsilon_{i}\right)$ is the concrete distribution relaxation of binary dropout as indicated in Equation (3). Finally, to achieve a sparse representation, the Molchanov et al. [37] approach was employed, in which a threshold τ is applied to the variational weights to force the sparse representation by $z={\left\{0,1\right\}}^{D}$.

### 2.2. Dataset Description

Throughout this study, the Instituto Nacional de Astrofísica, Óptica y Electrónica (INAOE) thermogram dataset [41], released in December 2019, was used. Currently, this dataset is the only publicly available thermogram database composed of samples from 167 volunteers: 122 diabetic and 45 nondiabetic subjects. This dataset was originally intended to study how the temperature is distributed in the plantar region among diabetic and nondiabetic subjects and how those differences can be measured. However, thermograms have been widely used for DFU detection at an early stage [18,42,43].

The INAOE dataset is slightly unbalanced toward diabetic cases. For this reason, our initial aim was to balance this dataset. Firstly, an additional group of healthy subjects was included; therefore, an extended and more balanced database was created by fusing the INAOE dataset with a private local dataset. Then, because this initial procedure did not suffice to achieve a balanced dataset, synthetic data were generated via synthetic minority oversampling technique (SMOTE) [44]. In this way, the number of samples was increased from 167 to 244, 122 per class.

#### Local Dataset

Infrared (IR) images were acquired using an affordable TE-Q1 Plus camera from Thermal Expert™ (i3system Inc., Daejeon, Republic of Korea), described in detail in a previous publication [45]. An acquisition campaign was carried out among healthy coworkers (N = 22) at IACTEC, composed of nine women and thirteen men [46]. For each subject, four images were acquired at T0, T5, T10, and T15. The first image (T0) was acquired as soon as the subject adopted a supine position barefoot. The subsequent images were acquired every 5 min up to 15 min while the subject was keeping their feet off the ground. An unconstrained protocol was used for the acquisition in a room with controlled luminosity, humidity, and ambient temperature (25 °C). Because the acquisition protocol for the INAOE database employed 15 min resting position to reach a state of thermodynamic equilibrium [41,47], only the images corresponding to T15, from the internal dataset, were employed to create the extended database. No specific standardization procedure was carried out to fuse the datasets despite the different ambient conditions and devices employed for the respective acquisitions.

### 2.3. Feature Extraction

**Figure 2:**
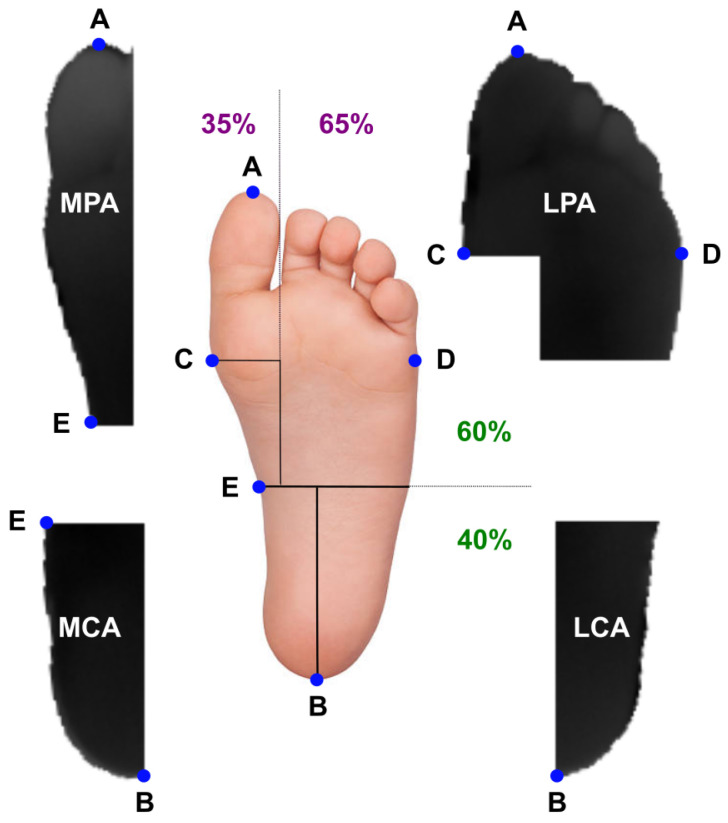
Graphical illustration of the defined angiosomes. The main reference points considered and the proportional foot division are also specified [41,47]. Reference point A was located at the tip of the innermost toe, whereas B at the center of the calcaneal base. Points C and D corresponded to the wider part of the foot. Point E corresponded to the 60% height of the foot.

Following the workflow proposed in the INAOE dataset, the IR images from the local dataset were processed to automatically segment the angiosomes, a composite unit of tissues supplied by an artery, as previously described in [41,47]. By considering these angiosomes, the foot was divided into four regions: Medial Plantar Artery (MPA), Lateral Plantar Artery (LPA), Medial Calcaneal Artery (MCA), and Lateral Calcaneal Artery (LCA), as illustrated in Figure 2.

This segmentation step was required to extract the features for each angiosome [48], which included the thermal change index (TCI) [47], the estimated temperature (ET), the estimated temperature difference (ETD), the hot spot estimator (HSE) [48], as well as the summarizing statistics (mean, standard deviation, maximum, minimum, skewness, and kurtosis). In addition, these features also extracted for the entire foot, and following previous approaches [18,48], a class, based on the normalized temperature ranges (NTR), was assigned to each foot.

Regarding the extraction of the TCI feature, despite the extended database containing more control subjects, the average control temperature was kept unchanged, being the values previously reported [41,47] considered as reference. These values are displayed in Table 1, and, for comparative purposes, the mean values corresponding to the healthy subjects, from the internal dataset, are also listed.

Furthermore, in order to extract the ET as well as the subsequently associated parameters, ETD and HSE, thermograms were clustered into classes based on temperature ranges. In the original study [48], Peregrina et al. used a dataset in which the feet were not segmented, so the background objects and their respective temperatures were present in the images. As a consequence, the classes were defined, from $C_{0}$ to $C_{7}$, whose temperatures were within the interval [25, 35) °C and, excluding $C_{0}$, each class covered 1 °C. To avoid high temperatures from heat sources unrelated to the feet, temperatures between [25, 28) °C were considered cold and associated with the background ($C_{0}$). The other classes were selected according to previously reported data [49], in which subjects with diabetes had a mean temperature of 30.2±1.3 °C, whereas for healthy subjects, the mean temperature was 26.8±1.8 °C. Because the dataset used in this work was previously segmented, the range of temperatures to be considered was extended, covering the interval [18, 37) °C; therefore, the classes were redefined as listed in Table 2. In this way, the complete range of temperatures in the dataset was taken into consideration. As can be observed, the number of classes was extended to 10, covering approximately 1 °C, except C${}_{1}$, C${}_{2}$, and C${}_{10}$. Furthermore, because the considered temperature interval was extended, the number of NTR classes was subsequently adjusted regarding the original study [18,48]. Finally, the mean value of the established intervals, the classmarks, were used for ET, ETD, and HSE feature extraction.

The nomenclature employed to name the aforementioned extracted features consisted of using a letter to specify the foot, ‘L’ for left and ‘R’ for right, followed by the name of the corresponding angiosome. For the features extracted using the entire foot, this second descriptor was discarded. Then, the variable was set using lowercase letters such as mean, std, max, min, skew, or kurtosis. Capital letters were employed for TCI, HSE, ET, and ETD, as well as for NTR followed by the subsequent class.

### 2.4. Feature Selection

Given a set of input variables $S=\{X_{1},X_{2},\ldots ,X_{n}\}$, a feature selection method aims to reduce the number of input variables to obtain a small subset of them that contains the most relevant and least redundant information about a desired variable *Y*. In this study, the number of input variables was as high as 188, and a detailed investigation was proposed to detect the most relevant features based on different approaches. These included some classical methods, random forest and LASSO, as well as two innovative ones based on variational DL (see Section 2.1).

Firstly, the original input set was optimized by removing highly correlated variables. The correlation estimation was carried out using the well-known Pearson correlation coefficient [50]; therefore, those features with a correlation r>0.95 were considered highly correlated. For instance, a high correlation between the mean value and the ET was observed, allowing a reduction in the number of features. Then, a feature ranking based on logistic regression was developed to select the most informative variables among them. These redundant variables were ranked based on an $AUC_{ROC}$ analysis [51] using the logistic model as an estimator. As a result, the number of features or input variables was reduced a ∼25.5%, from 188 to 140.

Five-fold cross-validation was employed, dividing the dataset into five folds (80% training and 20% testing set), and the performance metric for the testing set was computed five times. Therefore, around 196 samples were used for training and 48 for validation. The relevance of the features was the average value resulting from the five iterations during the cross-validation.

## 3. Results

**Figure 3:**
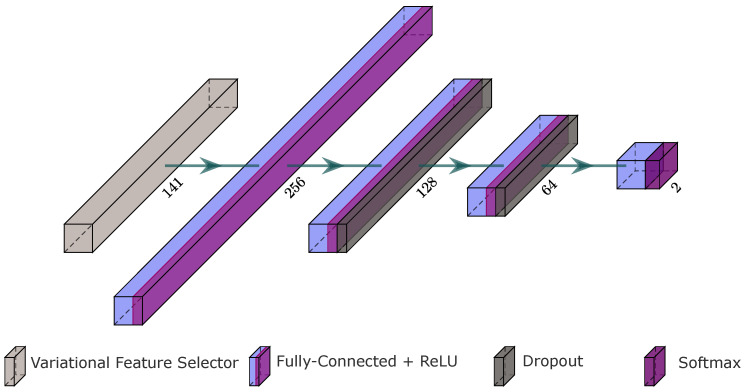
Deep learning architecture used for the feature selection based on variational dropout approaches. The first layer corresponds to variational dropout as a feature selector, illustrated in Figure 1.

Regarding the evaluation of feature ranking based on variational DL approaches, the implemented architecture is depicted in Figure 3. As can be observed, the variational feature selector was just used in the first layer after the input. Two dropout layers were added in the following layers to mitigate overfitting problems, in which a ρ=0.2 was employed as the dropout rate.

The results presented in this section were extracted using a batch size of 32 samples during the training process, having a minimum batch size of 4 in the last iteration. The ADAM optimizer [52] was used for training the DL model. The parameters to control exponential decay rates for the moment estimation, $\beta_{1}$ and $\beta_{2}$, were set to 0.9 and 0.999, respectively. The learning rate (lr) was set to $10^{-2}$ when the variational feature selector was the concrete dropout approach and $lr=10^{-3}$ when variational dropout was applied. The number of training epochs was set to 500.

In order to avoid many features becoming pruned early during the first iteration of the training, a Lagrange multiplier, λ∈[0,1], was employed in the regularization term of Equations (4) and (8). So, the model was able to learn a valuable representation of the data in the latent spaces before being heavily penalized. Specifically, λ linearly increases from 0 to 1 using a step size of $2.5\times 10^{-3}$ per epoch. This approach is based on the $D_{KL}$ annealing trick for variational autoencoders [34] previously proposed in [53].

**Figure 4:**
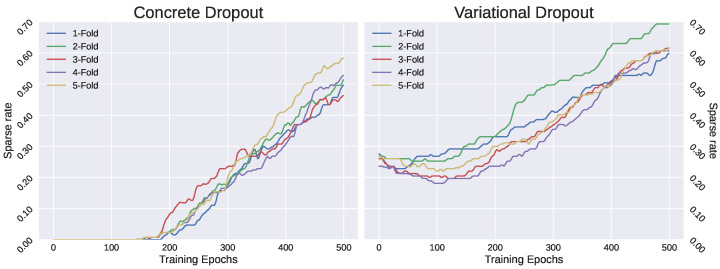
The sparse rate obtained in the variational feature selector using τ>0.9 as threshold in the different cross-validation iterations.

The performance of the models was evaluated by applying τ>0.9 (see Section 2.1.2) to obtain the sparse representation of the original input space. Figure 4 shows the sparse rate during the training phase of the respective model in each iteration of the cross-validation. As can be observed, concrete dropout obtained a sparse rate of around 50%, and the variational dropout approach obtained a sparse rate of around 60% in most of the cases. This means that, in general, more than half of the features were considered irrelevant. Additionally, variational dropout started to become sparser in an early epoch, whereas concrete dropout required a higher λ. According to the sparse representation, using the test set in each fold, average accuracies were 89.1% and 85.7% for concrete and variational dropout, respectively. In addition, we noticed that using the variational parameter, ϕ, as feature ranking, the most important features were roughly the same in all the experiments. In comparison, the LASSO approach received a sparse rate of 44%, using a lower number of features than the DL approaches, and with an approximate accuracy of 90%. These results were not reliable for comparison purposes because the models were fully optimized, including the hyperparameters, and the test set was not large enough to reject a possible overfitting.

### 3.1. Feature Selection

Following the workflow described in Section 2.4, the most relevant features, listed in Table 3, were extracted for all the approaches considered: LASSO, random forest, and concrete and variational dropout. For the LASSO approach, the feature ranking was estimated by the absolute value of its coefficient. In relation to concrete and variational dropout, the variational parameter was used as feature ranking (see Section 2.1.2).

The 10 first features extracted for each approach were considered the most relevant and are highlighted in bold in Table 3. Therefore, approximately 0.05% of the total features extracted were considered relevant. Regarding the distribution of these features by angiosome, MPA and LPA presented the largest number of features with a total of nine and six associated features, respectively. LCA and MCA angiosomes had, respectively, three and four associated features each. For the entire foot, only two associated features were found.

Furthermore, the ten first features found to appear in all the implemented approaches are listed by rank in Table 4. The ranks of these features changed according to the approach employed. Thus, the lowest rank of each feature, among the different approaches, was assigned as its final rank. The search for coincidence was restricted to the first 30 ranked features provided for each approach. However, as observed in Table 4, the assigned ranks are listed in intervals ranging from ten units. Features found up to a rank lower than 50 were considered. As noticed, if only the 10 first features in coincidence were considered, the angiosomes with more associated features were LPA and the entire foot, with three associated features both, whereas MCA and LCA had two associated features each. No associated features were found for the MPA angiosome in this case.

Considering the features in coincidence among the different approaches, Table A1, in Appendix A, depicts an extended version of the most promising features distributed per angiosome. As observed, the largest number of features in coincidence, a total of four, was associated with the LPA angiosome.

An SVM [32] classifier was used with all the features as input to provide a reference aiming to quantify the performance of the extracted features, their rank, and selected combination. SVM aims to generate a hyperplane in a high-dimensional space, generated by a kernel, that separates the data into classes. Initially, using the available features as input, the SVM classifier was optimized using a randomized search [54] to obtain the best parameters. As a result, a Gaussian kernel, also known as the radial basis function (RBF) kernel, was used. The RBF kernel has a hyperparameter, γ, that controls the spread of the Gaussian center. In addition, the hyperparameter *C* in SVM is used for directing the $L_{2}$ penalty, which controls the trade-off between decision boundary and misclassification. The best performance, displayed in Table 5, was achieved with a γ value of 0.0035 and a *C* value of 7.743.

### 3.2. Evaluation of Features by SVM Classifier

Several experimental settings were considered to evaluate the extracted features for the chosen classification task, which was to distinguish between healthy and diabetic patients. In this case, the SVM classifier was not optimized; that is, standard hyperparameters were chosen to offer a fair comparison between the proposed approaches to rank the features. For the different experiments described in this section, γ was set to 0.1, motivated by the low dimensional space of the input data. In addition, the hyperparameter *C* was set to 1. This configuration was the same for the different selected features, trying to avoid bias in the conclusions due to well-fitted settings for the indicated features. The average value resulting from five-fold cross-validation, testing the models five times, was used for the metrics estimation depicted in Table 6, as previously reported [18].

First, the SVM was fed with the ten first features extracted for each approach, LASSO, random forest, and concrete and variational dropout (features highlighted in bold in Table 3). Second, the ten first features in coincidence, this is, those that appeared in all the approaches and are listed in Table 4, were also employed to feed the classifier. Finally, to compare the features extracted and the subsequent classification task with those from a previous study [18], the following ten ranked features were also considered: TCI, NTR_C${}_{4}$, NTR_C${}_{3}$, MPA_mean, LPA_mean, LPA_ET, LCA_mean, highest temperature, NTR_C${}_{2}$, and NTR_C${}_{1}$. These features were among the top ten features resulting from testing several techniques, which included Pearson, chi square, recursive feature elimination (RFE), logistics, random forest, and LightGBM. The metrics of the performance for each approach, according to the experimental settings described, are listed in Table 6.

Notice that, contrary to the setup employed in the present study in which all features were extracted by foot, L or R, the foot to which the previously mentioned features were associated was not specified in [18]. Therefore, the mean value between both feet was calculated in order to match these features and offer a fair comparison. In addition, the NTR class definition considerably differed from the one considered previously; thus, the equivalent class, based on temperature values, was used instead. NTR_C${}_{4}$ and NTR_C${}_{3}$ in the original study corresponded to ranges between 31 and 32 °C as well as 30 and 31 °C, respectively [18,48]. In the present study, the closest approximations were NTR_C${}_{8}$ and NTR_C${}_{7}$, for which the respective ranges coincided with the ranges mentioned above.

Considering the features extracted for each approach and the subsequent classification task, all approaches provided good metric values. However, the best scores, except for the recall parameter, were observed for the concrete dropout approach. When the set of relevant features were those common to all the approaches, although at different rank positions, the performance in this experimental setting provided the best recall. Furthermore, as noticed, the recall values were lower in comparison with the other parameters of the performance metrics. This may have been due to the imbalance between healthy and diabetic samples from the original dataset, because a low recall score is associated with a high number of false negatives. A relevant number of healthy samples was generated for balancing using SMOTE, which performed a linear interpolation between samples. Therefore, recall was penalized because it was exclusively dependent on the diabetic samples. In this case, considering the precision–recall tradeoff, a lower recall was preferred due to the associated implications.

As shown in Table 6, the performance of all the models when using the corresponding first 10 features as well as when using the first 10 features in coincidence, was quite similar to those considered as reference values (shown in Table 5). However, the classical LASSO approach and DL-based concrete dropout exhibited a slightly better performance with only 10 features.

## 4. Discussion

Several approaches were considered to select relevant features used for DFU detection based on infrared thermograms. Classical approaches, LASSO and random forest, were tested versus two innovative approaches based on DL, concrete and variational dropout. The outputs of these approaches were analyzed to extract a new set of features considered relevant to classify whether a thermogram corresponded to a healthy or diabetic person. The results provided by the proposed approaches exhibited promising results, particularly for the concrete dropout approach.

Regarding the performance of the traditional approaches in comparison with that of the DL-based ones, LASSO provided results close to those of the concrete dropout, according to the F1 score, although the latter exhibited a slightly better performance compared with the established reference values. However, LASSO is limited to linear solutions, while concrete dropout does not suffer from this limitation. No fine-tuning of the models was implemented to increase the respective performance, because a comparison between extracted input features was intended. Thus, when a few features were used, i.e., 10, both methods produced promising performance. In this particular case, the LASSO approach would be an easy-to-implement and faster alternative to concrete dropout, as comparable performance was achieved. Furthermore, considering the most relevant features of each approach, six of the selected features matched for these two approaches, see Table 3. Thus, the similarity in performance may have been due to this coincidence of features.

For further comparison, the optimization of these two approaches, considering 10 input features, was performed. The hyperparameters considered for fine-tuning were the kernel (RBF, linear, or polynomial), the degree of the polynomial in case the corresponding kernel was selected, γ, and *C* (data not shown). For the LASSO approach, the best model used a third-degree polynomial kernel with a γ value of 0.1 and a *C* value of 2.2. The best concrete dropout settings were achieved for the RBF kernel, with a γ value of 0.3, and a *C* value of 3.8. The F1 scores were approximately 0.89 and 0.90 for LASSO and concrete dropout, respectively. Thus, the performance of the LASSO approach closely matched that of the reference (0.8965±0.0837). Additionally, an increase in the number of input features from 10 to 50, in combination with an optimized SVM, exhibited a slightly increased performance for the LASSO approach, with an F1 score of roughly 0.91. However, the performance of the concrete dropout decreased with an approximate F1 score of 0.87. In this case, both approaches used an RBF kernel, with γ being 0.06 and 0.04 and *C* being 1.2 and 1.5 for LASSO and concrete dropout, respectively. This improvement observed with LASSO when the number of features was increased might have been produced by the oversampling based on SMOTE, which generated 77 new samples by a linear interpolation between samples from the minority class. This process might have added correlation to the dataset, making it more likely that LASSO could find features with a high degree of correlation. Further analysis is planned to confirm this hypothesis. Regardless, concrete dropout is less sensitive to these problems due to its nature. In any case, these results were achieved by cross-validation, testing with around 48 samples per fold. In our previous study [55], using the INAOE dataset, we showed that the traditional classification metrics were not reliable due to the small amount of data in the test set, which might be a nonrepresentative subset to evaluate the model. On the other hand, the decrease observed in the performance of concrete dropout when the number of features was increased seemed plausible due to the implicit noise added by the extra features.

A previous study [18] was considered as a reference to quantify the performance of the extracted features for the classification task. This reference study employed a stacking classifier using gradient boost, XGBoost and random forest considering previously ranked features as input: TCI, NTR_C${}_{4}$, NTR_C${}_{3}$, MPA_mean, LPA_mean, LPA_ET, LCA_mean, and highest temperature. The best classification performance achieved was reported as approximately 94% accuracy, precision, sensitivity, and F1 score. Using these proposed features, the values reported in the present study, around 77% in the F1 score, are considerably lower than those reported previously (see Table 6). However, although the definition of the features was slightly modified and the classifier employed considerably differed; the same input features exhibited a roughly 15% lower performance in comparison with the features extracted in this study. This difference may be explained by the use of an extended dataset as well as their proposed new labeling in the INAOE dataset for distinguishing between mild, moderate, and severe cases in the diabetic foot domain, which was not tested in this study. Another state-of-the-art work recently reported, employing the INAOE dataset, extracting features by clusters instead of by angiosomes [56]. In addition, a new feature, the cluster thermal index (CTI), was proposed, which provides a measure of temperature deviation between a subject and the control group, considering not only the temperature difference between the clusters but also the range of temperature in the control group. In this case, several models were provided to classify healthy and diabetic subjects. Multiclassification was employed to refine the stratification of diabetic patients using logistic regression, SVM, and K-nearest neighbors. The results reported for the binary classification with SVM are comparable to those reported here, the accuracy being approximately 86%. Furthermore, using only 50 thermograms from the INAOE database and extracting texture features, the reported accuracy of the SVM classifier was roughly 96% [57]. In addition, employing a private dataset composed of 24 healthy and 36 diabetic subjects, a binary classification using SVM achieved 95% accuracy [17]. These values are quite superior to those reported in this paper. However, a true comparison cannot be drawn because the set of features employed considerably differed.

Before feature extraction, our initial study focused on establishing a balanced dataset of diabetic and healthy subjects by fusing a publicly unbalanced available dataset [41] with a local dataset composed of healthy subjects. Furthermore, the preprocessing of the thermograms was also carefully considered to extract the features previously reported [18,41]. Among set of features, considered within the state-of-the-art features, were the highest temperature, TCI, HSE, ET, NRT, and several statistical variables such as the mean value, being associated with the entire foot as well as some defined angiosomes (see Section 2.3). Notably, in the present stduy, these features were extracted by foot, and considered separately, unlike previous studies in which an average between the R and L foot was assumed due to the lack of specific information on the procedure. For this reason, the number of features extracted is considerably much higher than in previous reports [18], 188 versus 37 features.

Of the most important state-of-the-art features, TCI is especially relevant. The TCI is focused on providing a quantification of the thermal change, independent of the observed distribution, and a difference of 1 °C is considered enough to notice a significant difference between the classes proposed [47]. In this study, reference values were not modified, in comparison with the original study, to calculate the TCI values despite more healthy subjects being considered in the extended dataset. Regardless, none of the features related to TCI were considered relevant among all the implemented approaches or within those features in coincidence.

Regarding NTR, the number of classes based on the thermogram temperatures was extended to 10 because the range of relevant temperatures considered was increased from 18 to 37 °C compared wtih the original study, which was from 25 to 35 °C [48]. These modifications were motivated by, first, the exclusion of temperature values characteristic of healthy patients, which were excluded in the original study to avoid the background, because foot segmentation was not available. The private thermograms from healthy volunteers, fused with the INAOE database for balancing purposes, showed that the temperature distributions were below 28 °C for many subjects. Second, these private thermograms were previously segmented; thus, excluding other heat sources within the background was not required. As a result, we did not discard any NTR class as was proposed in [48] for removing the background. A better-performing classifier was expected by extending the temperature ranges. However, most of the features related to the NTR were not considered relevant and, similar to that observed for the TCI, none were obtained within the features in coincidence among all the approaches. Furthermore, according to the F1 score, the best-performing approach was concrete dropout, and not a single feature related to the NTR was among those considered relevant.

Opposite to that described above, in the present study, among those specially designed features for DFU, only HSE and ETD seem to be relevant. Furthermore, as described, the sole of the foot was divided into four different angiosomes, and their individual features were extracted. The extraction demonstrated an unbalanced significance of the angiosomes and, therefore, the division of the foot into angiosomes seemed a determinant factor for feature extraction and played an important role in the analysis. In particular, the LPA angiosome appeared as the most predictive, with more associated features than the other angiosomes, followed by LCA (see Table A1).

Perhaps the extended dataset employed in this study, based on two different population samples, added a varying contribution of pathogenic factors that led to variable outcomes [58]. The present study can be considered sort of a multicenter study, providing a generalization factor for the classification task at hand, and therefore, the set of relevant features may be significantly changed from previous studies. Further studies are required with an increased dataset, composed of a balanced number of diabetic and healthy subjects, and preferably from different population samples, in order to continue generalizing the existing approaches. Furthermore, we will continue toward the study of the importance of the different angiosomes as well as the exploration of new, interesting features that appear within the state-of-the-art methods. Most importantly, the assessment of their predictive value for classification will also be an area to explore in detail.

## 5. Conclusions

An extended dataset was employed to extract relevant features from infrared thermograms to be used for DFU detection in order to classify whether a subject is healthy or diabetic. For feature extraction, two classical approaches, LASSO and random forest, were tested versus two innovative approaches based on DL, concrete and variational dropout. The final set of features extracted substantially differed from those considered within the state-of-the-art methods. The same SVM classifier employed to quantify the performance of the new set of features extracted in this study provided approximately 15% better performance than those features previously reported as relevant.

## Figures and Tables

**Table 1:** Mean temperature values per angiosome in the control group for the INAOE database [47] and the local dataset described in Section 2.2. SD indicates standard deviation.

Angiosome	INAOE		Local	
	$\bar{T}$ (°C)	SD	$\bar{T}$ (°C)	SD
MPA	25.8	1.4	25.0	3.0
LPA	25.7	1.3	24.5	3.1
MCA	26.4	1.3	24.5	2.5
LCA	26.1	1.4	24.5	2.6

**Table 2:** Classes defined according to the temperature of the foot’s thermograms.

Class	NTR Class	Interval (°C)	Classmark (°C)
C${}_{1}$	NTR_C${}_{1}$	[18,22)	20.0
C${}_{2}$	NTR_C${}_{2}$	[22,26)	24.0
C${}_{3}$	NTR_C${}_{3}$	[26,27)	26.5
C${}_{4}$	NTR_C${}_{4}$	[27,28)	27.5
C${}_{5}$	NTR_C${}_{5}$	[28,29)	28.5
C${}_{6}$	NTR_C${}_{6}$	[29,30)	29.5
C${}_{7}$	NTR_C${}_{7}$	[30,31)	30.5
C${}_{8}$	NTR_C${}_{8}$	[31,32)	31.5
C${}_{9}$	NTR_C${}_{9}$	[32,33)	32.5
C${}_{10}$	NTR_C${}_{10}$	[33,37)	35.0

**Table 3:** The 30 most relevant features extracted, listed according to rank, for all the approaches considered: LASSO, random forest, and concrete and variational dropout. The 10 first features are highlighted in bold as the most relevant for each method. The nomenclature employed is defined in Section 2.3.

Rank	LASSO	Random Forest	Concrete Dropout	Variational Dropout
1	**R_LPA_min**	**L_MPA_min**	**R_LPA_min**	**R_LPA_min**
2	**L_LPA_std**	**R_LPA_min**	**R_MCA_std**	**R_MPA_HSE**
3	**Foot_ETD**	**L_MPA_NTR_C${}_{3}$**	**Foot_ETD**	**MCA_ETD**
4	**L_MPA_min**	**R_MCA_std**	**R_LCA_kurtosis**	**L_kurtosis**
5	**L_MPA_skew**	**R_LPA_std**	**R_LPA_std**	**L_MPA_skew**
6	**L_LCA_NTR_C${}_{4}$**	**L_MPA_std**	**L_MPA_min**	**L_MCA_skew**
7	**R_LPA_NTR_C${}_{3}$**	**L_LPA_NTR_C${}_{2}$**	**LPA_ETD**	**R_LCA_kurtosis**
8	**R_MPA_NTR_C${}_{4}$**	**L_LPA_std**	**L_LPA_std**	**R_LPA_std**
9	**L_MPA_HSE**	**R_LCA_NTR_C${}_{2}$**	**L_MCA_skew**	**L_MPA_NTR_C${}_{4}$**
10	**R_MCA_std**	**R_MPA_NTR_C${}_{2}$**	**L_MPA_HSE**	**L_LCA_std**
11	LCA_ETD	R_MPA_std	R_LCA_skew	R_LPA_HSE
12	MCA_ETD	R_LCA_mean	MCA_ETD	R_LCA_std
13	R_LCA_kurtosis	L_MCA_min	L_MCA_std	Foot_ETD
14	R_MPA_NTR_C${}_{3}$	L_LCA_NTR_C${}_{2}$	MPA_ETD	R_MCA_std
15	R_LCA_NTR_C${}_{3}$	L_MCA_mean	LCA_ETD	LPA_ETD
16	L_kurtosis	R_MPA_ET	R_MCA_skew	L_MCA_std
17	R_std	R_std	L_MPA_skew	R_MCA_skew
18	R_LCA_HSE	L_MCA_NTR_C${}_{2}$	R_kurtosis	L_MCA_NTR_C${}_{5}$
19	R_skew	L_LPA_ET	R_HSE	R_MCA_HSE
20	L_HSE	L_LPA_NTR_C${}_{1}$	L_LCA_kurtosis	R_kurtosis
21	R_LPA_NTR_C${}_{5}$	L_LCA_NTR_C${}_{3}$	R_LCA_std	LCA_ETD
22	L_max	Foot_ETD	L_MPA_std	R_LCA_skew
23	L_MCA_std	LPA_ETD	L_LCA_std	R_MPA_std
24	L_LPA_NTR_C${}_{4}$	L_MPA_NTR_C${}_{4}$	R_LCA_HSE	R_MPA_NTR_C${}_{4}$
25	L_MCA_NTR_C${}_{3}$	L_NTR_C${}_{3}$	R_skew	L_MPA_NTR_C${}_{3}$
26	LPA_ETD	L_std	R_MPA_NTR_C${}_{4}$	L_LPA_NTR_C${}_{2}$
27	R_MCA_HSE	L_kurtosis	R_MPA_HSE	R_LCA_HSE
28	L_MCA_skew	R_MPA_NTR_C${}_{3}$	L_MCA_kurtosis	L_MPA_HSE
29	L_LPA_NTR_C${}_{5}$	L_MCA_std	L_LCA_NTR_C${}_{4}$	L_MCA_HSE
30	R_NTR_C${}_{5}$	L_LCA_max	L_kurtosis	L_skew

**Table 4:** Most relevant features that coincided in all the approaches considered, listed according to rank.

Rank	Features in Coincidence
Rank < 10	R_LPA_min
Rank < 20	R_MCA_std
Rank < 30	Foot_ETD
	LPA_ETD
	L_MCA_std
	L_kurtosis
Rank < 50	L_LPA_std
	R_kurtosis
	R_LCA_std
	R_LCA_kurtosis

**Table 5:** Performance metrics of the optimized SVM classifier using all available features as input.

Input Dataset	Accuracy	Precision	Recall	F1 Score
All features	0.9099 ± 0.0613	0.9473 ± 0.0705	0.8535 ± 0.1016	0.8965 ± 0.0837

**Table 6:** Performance metrics of the approaches considered, according to the selected input features, in each experimental setting. The highest value for each performance metric is highlighted in bold.

Input Dataset	Approach	Accuracy	Precision	Recall	F1 Score
First 10 features	LASSO	0.8975 ± 0.073	0.9533 ± 0.079	0.8361 ± 0.130	0.8908 ± 0.107
	Random Forest	0.8893 ± 0.070	0.9703 ± 0.080	0.8033 ± 0.118	0.8789 ± 0.103
	Concrete dropout	**0.9098 ± 0.069**	**0.9808 ± 0.057**	0.8361 ± 0.131	**0.9027 ± 0.104**
	Variational dropout	0.8934 ± 0.054	0.9615 ± 0.049	0.8197 ± 0.104	0.8850 ± 0.081
First 10 features in coincidence	LASSO, random forest, concrete and variational dropout	0.9057 ± 0.066	0.9626 ± 0.052	**0.8442 ± 0.135**	0.8995 ± 0.102
Selected features from [18]	Pearson, chi square, RFE, logistics, random forest, and LightGBM	0.7951 ± 0.075	0.8750 ± 0.136	0.6885 ± 0.089	0.7706 ± 0.103

## Data Availability

The data presented in this study are available on request. The data are not publicly available due to privacy restrictions. The code implemented for this study is freely available at https://github.com/mt4sd/DFUFeatureRankingByVariationalDropout (accessed on 2 January 2023).

## Appendix A

Features in coincidence among the different feature-selection methods are listed, according to angiosome, in the following table.

**Table A1:** Most relevant features extracted for all the approaches considered and listed according to angiosome. The search for coincidence was restricted to the first 30 ranked features provided for each approach. Features found up to a rank lower than 50 were considered.

Region	Features
MPA	R_MPA_HSE
	L_MPA_skew
LPA	R_LPA_min
	LPA_ETD
	L_LPA_std
	R_LPA_std
MCA	R_MCA_std
	L_MCA_std
LCA	R_LCA_std
	R_LCA_kurtosis
	LCA_ETD
Foot	Foot_ETD
	L_kurtosis
	R_kurtosis
